# Does marital status impact postoperative survival in patients with less differentiated hepatocellular carcinoma? A population‐based study

**DOI:** 10.1002/cam4.2536

**Published:** 2019-09-02

**Authors:** Bing Yan, Dou‐Sheng Bai, Jian‐Jun Qian, Chi Zhang, Sheng‐Jie Jin, Guo‐Qing Jiang

**Affiliations:** ^1^ Department of Hepatobiliary Surgery Clinical Medical College Yangzhou University Yangzhou China; ^2^ Department of Hepatobiliary Surgery The Second Clinical College Dalian Medical University Dalian China

**Keywords:** hepatocellular carcinoma, less differentiated, marital status, SEER, survival

## Abstract

**Background:**

Marital status has long been widely recognized as a determinant of cancer survival. However, only few analytical studies have been conducted on this issue considering heterogeneous factors. The aim of this study was to assess the effects of marital status on postoperative survival of patients with less differentiated (poor/anaplastic) hepatocellular carcinoma (HCC).

**Methods:**

We retrospectively analyzed 1581 postoperative patients diagnosed with poor/anaplastic HCC from the Surveillance, Epidemiology, and End Results database between 2004 and 2015. Patients were classified into married, never married, divorced/separated and widowed groups. Kaplan‐Meier analysis and multivariate Cox regression analysis were used to analyze the effects of marital status on HCC cause‐specific survival (HCSS).

**Results:**

Compared with married patients, there were no significant differences in HCSS for unmarried, never married, divorced/separated and widowed patients both in univariate (5‐year HCSS: 36.0% vs 36.3%, 36.0% vs 32.4%, 36.0% vs 40.2%, 36.0% vs 36.3%, respectively, all *P *> .05) and multivariate analysis (all *P *> .05). Furthermore, in stratified analyses according to sex, age, and tumor size, compared with married patients, there were also no significant differences for never married, divorced/separated, and widowed patients both in univariate (all* P *> .05) and multivariate analysis (all *P *> .05).

**Conclusions:**

For patients with poor/anaplastic differentiated HCC treated with surgical resection, marital status has no prognostic role in survival.

## Graphical abstract

Although marital status has long been widely recognized as a determinant of cancer survival, only few analytical studies have been conducted on this issue considering heterogeneous factors. This study found that marital status has no prognostic role in survival of patients with less (poor/anaplastic) differentiated hepatocellular carcinoma treated with surgical resection.

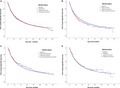

## 1 INTRODUCTION

Hepatocellular carcinoma (HCC) is the fifth most common cancer and the third cause of cancer‐related mortality worldwide.^1^ More than half of the new cases and deaths associated with liver cancer globally occur in China every year.^2^ The current data in China show that HCC has the fourth highest morbidity and third highest mortality rates among all cancers.^3^ Although some treatments have been applied over the past few decades, including radiotherapy and chemotherapy, the overall survival rate has increased only slightly, and the prognosis of HCC remains generally low, with the 1‐year survival rate <50%.^4^

Marital status has long been widely related to cancer mortality rates. Extensive studies have indicated that married patients have better survival outcomes compared with unmarried patients in almost all organ cancers, such as head and neck cancer,^5^ breast cancer,^6^ esophageal cancer,^7^ pancreatic cancer,^8^ gastric cancer,^9^ small intestinal adenocarcinoma,^10^ renal cell carcinoma,^11^ bladder urothelial carcinoma,^12^ testis cancer,^13^ cervical cancer,^14^ and Hodgkin lymphoma.^15^ A recent large population‐based study showed that marital status has a prognostic role in postoperative survival in HCC (*P* < .001).^16^ However, only few analytical studies have been focused on this issue considering some heterogeneous factors. Pathological grade may be a heterogeneous clinical variable that impacts the prognostic capability of marital status on survival. To date, the effect of marital status on postoperative patients with less differentiated (poor/anaplastic) HCC has not been well studied. Therefore, in the present study, we analyzed whether marital status affects survival outcome in postoperative patients with less differentiated HCC from the Surveillance, Epidemiology, and End Results (SEER) database.^17^

## 2 MATERIALS AND METHODS

### 2.1 Data source

Research data were extracted from the SEER database (http://www.seer.cancer.gov; Incidence—SEER 18 Regs Research Data + Hurricane Katrina Impacted Louisiana Cases, Nov 2017 Sub [1973‐2015 varying]). Data included patient demographics, primary tumor site, tumor size, stage at diagnosis, radiotherapy, chemotherapy and survival, which were regularly recorded in the SEER registries and assessed the impact of cancer in the general population. The SEER program is a reliable source of cancer morbidity and survival in the United States.

### 2.2 Patient selection

We used SEER*Stat 8.3.5 software to extract information of patients diagnosed with HCC between 2004 and 2015. Patients who met the following criteria were included: (a) treatment with surgical resection, (b) pathological type was less differentiated (poor/anaplastic), and (c) histological codes were limited to HCC (8170, 8171, 8172, 8173, 8174, and 8175). Patients who met the following criteria were excluded: (a) unknown marital status or domestic partner (n = 2), and (b) no first tumor. Finally, 1581 postoperative patients with poor/anaplastic HCC were extracted from the SEER database.

### 2.3 Study variables

Study variables included sex, race, age at diagnosis, year of diagnosis, TNM stage, tumor size, radiotherapy, chemotherapy, marital status, survival months and vital status. To obtain more patients extracted from the SEER database, we used the sixth edition TNM classification system defined by the AJCC Cancer Staging Manual. Patient race was defined as white, black, or other (American Indian/Alaskan Native, Asian/Pacific Islander and unknown); patients were categorized into two age groups: ≤60 years old and ＞60 years old; the radiotherapy patients included two groups: no and yes; the chemotherapy patients included two groups: no/unknown and yes; and marital status was classified as “married”, “single (never married)”, “divorced/separated”, and “widowed”. The “unmarried” were defined as the combination group of “widowed”, “single (never married)”, and “divorced/separated”.

### 2.4 Statistical analyses

In previous studies, married patients had the highest survival rate.^5^, ^6^, ^7^, ^8^, ^9^, ^10^, ^11^, ^12^, ^13^, ^14^, ^15^, ^16^ Therefore, we used married groups as references for comparison with the other four marital groups respectively, thus generating four subgroup outcomes. The baseline characteristics were presented as frequency (%) and were assessed using the chi‐square (*χ* ^2^) test of descriptive statistics. Survival differences were compared between groups using the Kaplan‐Meier analysis. All the prognostic factors associated with *P* < .05 in Kaplan‐Meier analysis were analyzed using multivariate Cox regression analysis. For subgroup analysis, in order to simply and intuitively describe the statistical results, we generated two forest plots using GraphPad Prism.^8^ HCC cause‐specific survival (HCSS) was the primary objective of this study. A *P* < .05 was considered statistically significant. All analyses were performed using statistical software IBM SPSS Statistics, Version 25.0 (SPSS, Inc).

## 3 RESULTS

### 3.1 Patient baseline characteristics

This study included 1581 eligible patients with HCC from 2004 to 2015, including 1159 (73.3%) male and 422 (26.7%) female patients. Of these patients, 1007 (63.7%) were married, 269 (17.0%) were never married, and 210 (13.3%) were divorced/separated, 95 (6.0%) were widowed. The married group had the highest proportion of men (76.9%), more prevalence of white patients (55.7%), and more stage I/II tumors (68.4%). Furthermore, women accounted for a large proportion in the widowed group (62.1%) compared with other marital groups. A considerable proportion of patients in the overall group did not receive radiotherapy (96.8%). Moreover, a large proportion did not receive chemotherapy (73.4%). Patient demographics and pathological features are shown in Table 1.

**Table 1:** Baseline demographic and clinical characteristics of HCC patients in SEER database

Characteristic	Total	Married	Never married	Divorced/Separated	Widowed	*P*
	n = 1581	n = 1007 (63.7)	n = 269 (17.0)	n = 210 (13.3)	n = 95 (6.0)	
	N (%)	N (%)	N (%)	N (%)	N (%)	
Sex						<.001
Male	1159 (73.3)	774 (76.9)	201 (74.7)	148 (70.5)	36 (37.9)	
Female	422 (26.7)	233 (23.1)	68 (25.3)	62 (29.5)	59 (62.1)	
Race						<.001
White	881 (55.7)	531 (52.7)	156 (58.0)	139 (66.2)	55 (57.9)	
Black	194 (12.3)	73 (7.2)	69 (25.7)	40 (19.0)	12 (12.6)	
Other^a^	506 (32.0)	403 (40.0)	44 (16.4)	31 (14.8)	28 (29.5)	
Age						<.001
≤60	754 (47.7)	462 (45.9)	179 (66.5)	96 (45.7)	17 (17.9)	
>60	827 (52.3)	545 (54.1)	90 (33.5)	114 (54.3)	78 (82.1)	
Year of diagnosis						.090
2004‐2007	482 (30.5)	331 (32.9)	74 (27.5)	53 (25.2)	24 (25.3)	
2008‐2011	530 (33.5)	336 (33.4)	94 (34.9)	66 (31.4)	34 (35.8)	
2012‐2015	569 (36.0)	340 (33.8)	101 (37.5)	91 (43.3)	37 (38.9)	
TNM Stage						.043
I/II	1081(68.4)	683 (67.8)	180 (66.9)	147 (70.0)	71 (74.7)	
III/IV	447 (28.3)	297 (29.5)	80 (29.7)	53 (25.2)	17 (17.9)	
Unknown	53 (3.4)	27(2.7)	9 (3.3)	10 (4.8)	7 (7.4)	
Tumor Size						.121
<3 cm	370 (23.4)	224 (22.2)	66 (24.5)	55 (26.2)	25 (26.3)	
3‐5 cm	472 (29.9)	290 (28.8)	80 (29.7)	74 (35.2)	28 (29.5)	
>5 cm	687 (43.5)	461 (45.8)	112 (41.6)	72 (34.3)	42 (44.2)	
Not stated	52 (3.3)	32 (3.2)	11 (4.1)	9 (4.3)	0 (0.0)	
Radiotherapy						.637
No	1530 (96.8)	972 (96.5)	261 (97.0)	203 (96.7)	94 (98.9)	
Yes	51 (3.2)	35 (3.5)	8 (3.0)	7 (3.3)	1 (1.1)	
Chemotherapy						.014
No/unknown	1160 (73.4)	740 (73.5)	180 (66.9)	163 (77.6)	77 (81.1)	
Yes	421 (26.6)	267 (26.5)	89 (33.1)	47 (22.4)	18 (18.9)	

Abbreviations: HCC, hepatocellular carcinoma; SEER, Surveillance, Epidemiology, and End Results; TNM, Tumor Node Metastasis.

a Other includes American Indian/AK Native, Asian/Pacific Islander) and unknowns.

### 3.2 Effect of marital status on HCC specific survival

**Figure 1:**
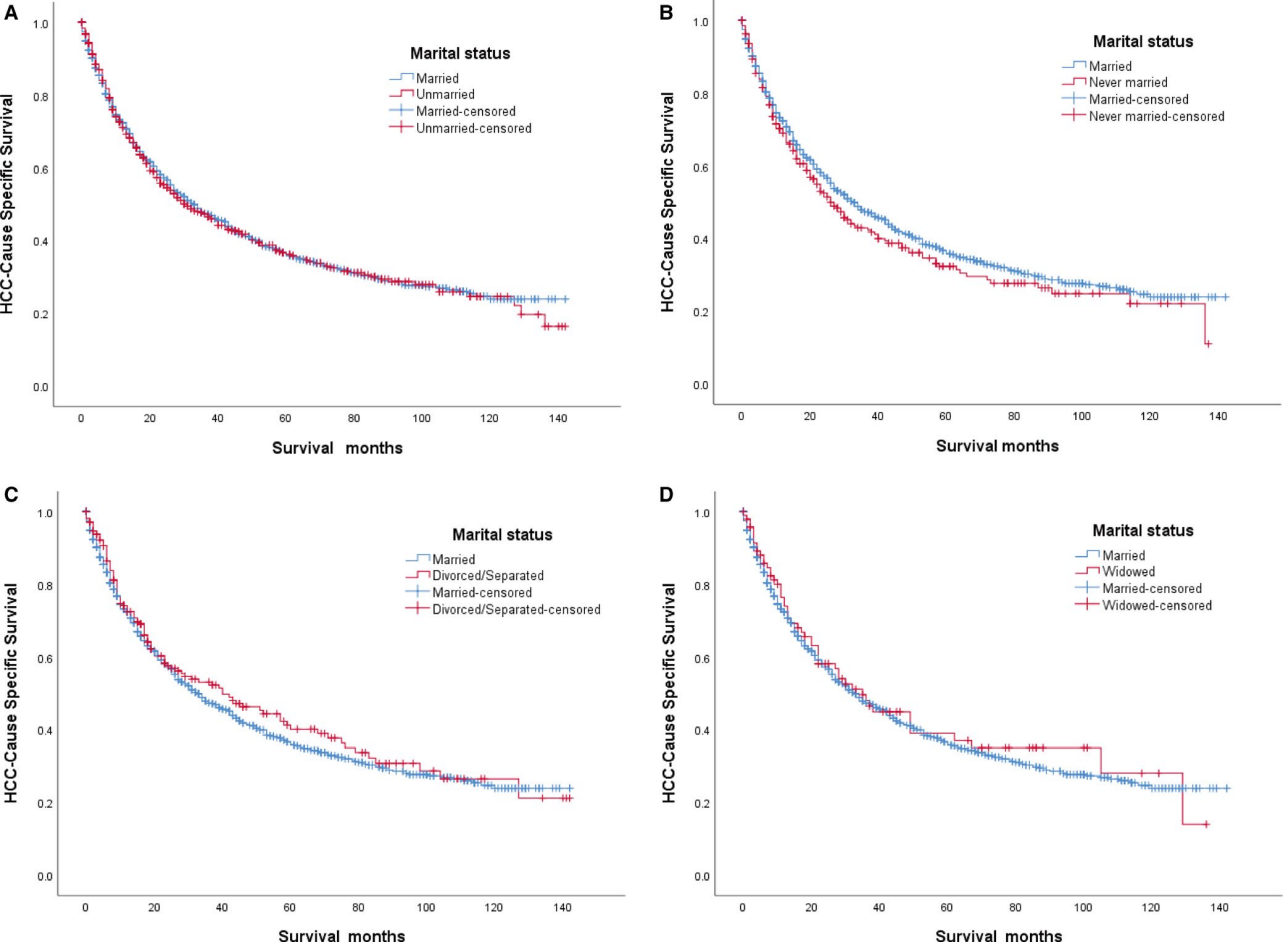
Kaplan‐Meier cancer‐specific survival curve of hepatocellular carcinoma patients according to marital status: (A) married and unmarried group: Log rank *χ* ^2^ test = 0.043, *P* = .836; (B) married and never married group: Log rank *χ* ^2^ test = 1.708, *P* = .191; (C) married and divorced/separated group: Log rank *χ* ^2^ test = 0.625, *P* = .429; (D) married and widowed group: Log rank *χ* ^2^ test = 0.208, *P* = .648

Race, sex, TNM stage, tumor size, radiotherapy, and chemotherapy were regarded as significant risk factors for survival on univariate analysis. These factors were analyzed using multivariate Cox regression analysis that showed race, sex, TNM stage, tumor size, and radiotherapy were independent prognostic factors for survival, but marital status was not an independent prognostic factor for survival (Table 2). We conducted Kaplan‐Meier analysis to analyze the difference in HCSS according to marital status. Overall, married patients showed no significant survival advantage compared with the unmarried patients (5‐year HCSS: 36.0% vs 36.3%, *P* = .836; Figure 1A). As shown in Figure 1 and Table 2. Compared with married patients, there were no significant survival differences for never married, divorced/separated and widowed patients in HCSS (5‐year HCSS: 36.0% vs 32.4%, *P* = .191, Figure 1B; 36.0% vs 40.2%, *P* = .429, Figure 1C; 36.0% vs 36.3%, *P* = .648, Figure 1D; respectively). Further, compared with married patients, multivariate Cox regression analysis showed that there were no significant survival differences for never married, divorced/separated and widowed patients (Hazard ratio [HR]: 1.075, 95% confidence interval (CI): 0.895‐1.291, *P* = .439; HR: 0.968, 95% CI: 0.785‐1.195, *P* = .764; HR: 1.110, 95% CI: 0.828‐1.489, *P* = .485; respectively).

**Table 2:** Univariate and multivariate survival analysis of HCSS in SEER database

Variable	Total	5‐y HCSS	Univariate analysis	*P*	Multivariate analysis	*P*
			Log rank *χ* ^2^ test		HR (95%CI)	
Sex			10.327	.001		<.001
Male	1159	33.7%			Reference	
Female	422	42.6%			0.711 (0.607‐0.834)	
Race			8.679	.013		.028
White	881	35.6%			Reference	
Black	194	29.8%			1.092 (0.888‐1.343)	.403
Other a	506	39.1%			0.837 (0.719‐0.974)	.021
Age			1.109	.292		NI
≤60	754	37.8%				
>60	827	34.1%				
Year of diagnosis			4.340	.114		NI
2004‐2007	482	33.8%				
2008‐2011	530	36.3%				
2012‐2015	569	46.7%				
TNM Stage			140.419	<.001		<.001
I/II	1081	43.2%			Reference	
III/IV	447	20.2%			1.733 (1.478‐2.032)	<.001
Unknown	53	23.6%			1.393 (0.957‐2.029)	.084
Tumor Size			130.216	<.001		<.001
<3cm	370	55.7%			Reference	
3‐5cm	472	39.3%			1.473 (1.204‐1.802)	<.001
>5cm	687	24.7%			2.003 (1.641‐2.446)	<.001
Not stated	52	11.2%			2.562 (1.759‐3.730)	<.001
Radiotherapy			8.938	.003		.027
No	1530	36.8%			Reference	
Yes	51	9.2%			1.479 (1.045‐2.093)	
Chemotherapy			6.021	.014		.716
No/unknown	1160	39.1%			Reference	
Yes	421	28.3%			1.028 (0.888‐1.189)	
Marital status			3.145	.370		.742
Married	1007	36.0%			Reference	
Never married	269	32.4%			1.075 (0.895‐1.291)	.439
Divorced/Separated	210	40.2%			0.968 (0.785‐1.195)	.764
Widowed	95	36.3%			1.110 (0.828‐1.489)	.485

Abbreviations: HCSS, hepatocellular carcinoma cause‐specific survival; SEER, Surveillance, Epidemiology, and End Results; TNM, Tumor Node Metastasis; NI, not included in the multivariate survival analysis.

a Other includes American Indian/AK Native, Asian/Pacific Islander and unknowns.

### 3.3 Subgroup analysis by sex, age, and tumor size

**Figure 2:**
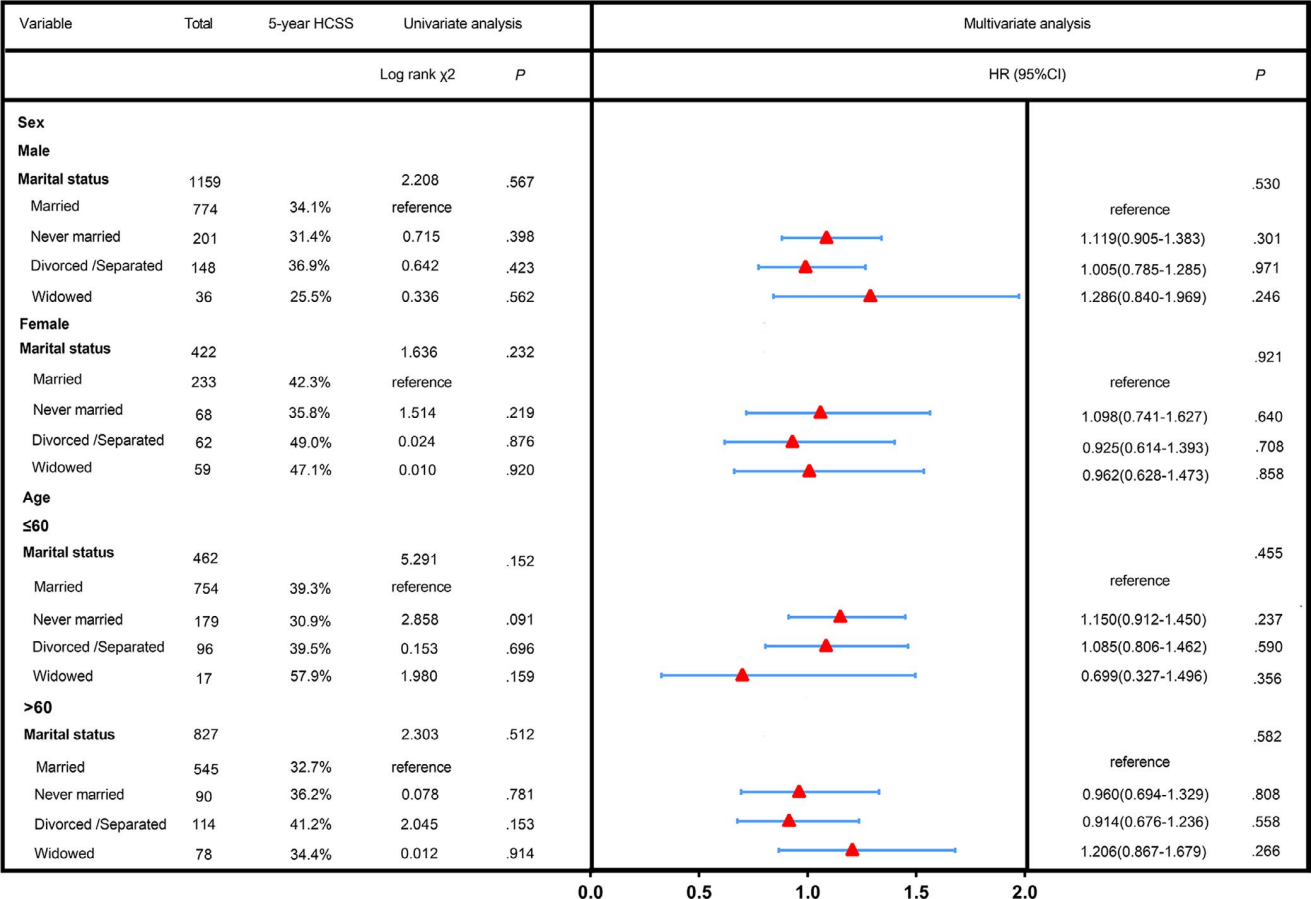
Univariate analysis and forest plot of the hazard ratio of hepatocellular carcinoma cause‐specific survival based on sex and age

**Figure 3:**
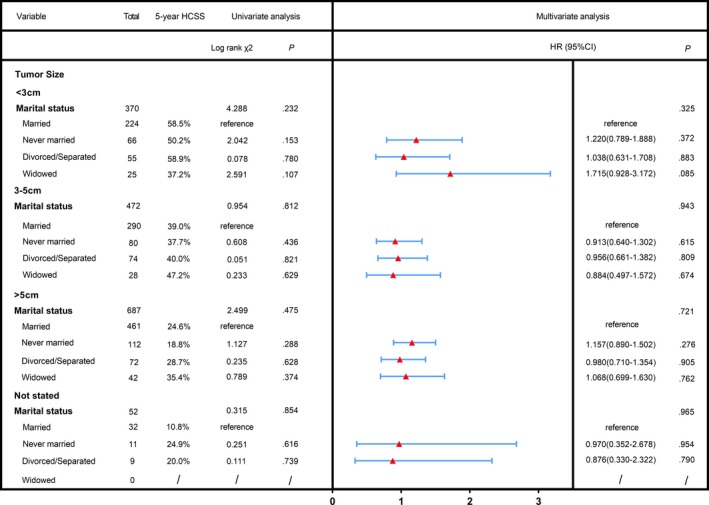
Univariate analysis and forest plot of the hazard ratio of hepatocellular carcinoma cause‐specific survival based on tumor size

As shown in Figure 2, marital status has no prognostic meaning for survival in the subgroups of sex and age both in univariate and multivariate analysis (all *P* > .05). Figure 3 shows the results for the subgroup analysis of the influence of marital status on survival in each tumor size both in univariate and multivariate analysis (all *P* > .05). These results indicated that marital status was not an independent prognostic factor for patients with less differentiated HCC treated with surgical resection.

## 4 DISCUSSION

It has long been widely recognized that marital status was an independent prognostic factor for survival in many organ tumors.^5^, ^6^, ^7^, ^8^, ^9^, ^10^, ^11^, ^12^, ^13^, ^14^, ^15^, ^16^ These data indicated that married patients had better survival in multiple cancers compared with unmarried patients. However, the majority of these studies included heterogeneous cohorts of patients, thus preventing an appropriate assessment of the usefulness of married status as a prognostic tool in a well‐defined subset of patients. The possible heterogeneous clinical variables, including pathological type, pathological grade, and so on, may be problematic. Regarding the effect of heterogeneity of pathological type, Jatoi et al used the Mayo Clinic database and revealed that the survival differences between married, single, divorced, and widowed patients with non‐small cell lung cancer were not significant.^18^ Taking into account the effect of heterogeneity of pathological grade, we think that patients with less (poor/anaplastic) differentiated HCC at diagnosis likely represent a specific group having significant heterogeneity to patients with well/moderately differentiated tumors at diagnosis. This subgroup appears to have more malignant biological behavior than those with well/moderately differentiated tumors, leading to a faster rate of progression and poorer survival. In our study, in considering the heterogeneity of different pathological grades and pathological types of liver cancer, we focused on less differentiated (poor/anaplastic) HCC. Notably, we found that marital status was not an independent prognostic factor for survival, and compared with married patients, there were no significant survival differences for never married, divorced/separated, and widowed patients in both univariate and multivariate Cox analyses. In addition, subgroup analysis according to sex, age, and tumor size showed that marital status has no prognostic meaning for survival in univariate and multivariate analysis. These conclusions are worthy of further exploration.

One study reported that married patients may benefit from spouse care and a better financial situation, which may be conducive to better survival.^19^ In contrast, unmarried, especially, widowed patients, may lack emotional and social support and may experience more distress, depression and anxiety than married counterparts, contributing to poor survival.^19^, ^20^ However, less differentiated HCC, which likely represents a cancer with more malignant biological behavior, may play a primary role influencing survival. Thus, in this setting, the effect of the advantages of married patients and the disadvantages of unmarried patients may be negligible. This reason may explain the contradictory results in our report compared with previous studies.^5^, ^6^, ^7^, ^8^, ^9^, ^10^, ^11^, ^12^, ^13^, ^14^, ^15^, ^16^

This study has several limitations. First, even if unmarried, some patients may have a live‐in partner, although the percentage of Americans who make such arrangements may be small. Such patients would be categorized as unmarried by SEER, which would likely cause deviations in our results. Second, only the marital status at diagnosis was recorded in the SEER database and this status may have changed after diagnosis. The changed marital status could also affect survival. Third, some information regarding smoking and alcohol also was not recorded in the SEER database, and it is possible that the absence of these variables may affect survival. Finally, information on socioeconomic status, education and quality of marriage is not available in SEER and thus was not included in our analysis, which slightly may bias our results. Therefore, given these limitations, our results should be cautiously interpreted.

In summary, for poor/anaplastic differentiated HCC patients treated with surgical resection, the 12‐year population‐based study shows that marital status has no prognostic role in survival. Our results are contrary to previous studies and may have important clinical significance that the heterogeneity of some determinants should be considered and defined within a narrow range as inclusion criteria in cancer survival studies.

## CONFLICT OF INTEREST

The authors have no conflicts of interest to disclose.

## AUTHOR CONTRIBUTIONS

Bing Yan: conceptualization, investigation, writing, visualization. Dou‐Sheng Bai: Conceptualization, investigation, writing, and visualization. Jian‐Jun Qian: investigation, data curation, and writing. Chi Zhang: formal analysis, data curation, resources, and writing. Sheng‐Jie Jin: Software, data curation, data linkage, and validation. Guo‐Qing Jiang: Conceptualization, data curation, formal analysis, funding acquisition, investigation, methodology, project administration, software, resources, supervision, writing‐original draft, and writing‐review and editing.

## Data Availability

These data were derived from the following resources available in the public domain: the Surveillance, Epidemiology, and End Results database (http://www.seer.cancer.gov; Incidence—SEER 18 Regs Research Data+Hurricane Katrina Impacted Louisiana Cases, Nov 2017 Sub [1973‐2015 varying]).

## References

[1] Kamangar F , Dores GM , Anderson WF . Patterns of cancer incidence, mortality, and prevalence across five continents: defining priorities to reduce cancer disparities in different geographic regions of the world. J Clin Oncol. 2006;24:2137‐2150. 16682732 10.1200/JCO.2005.05.2308

[2] Torre LA , Bray F , Siegel RL , Ferlay J , Lortet‐Tieulent J , Jemal A . Global cancer statistics, 2012. CA Cancer J Clin. 2015;65:87‐108. 25651787 10.3322/caac.21262

[3] Chen W , Zheng R , Baade PD , et al. Cancer statistics in China, 2015. CA Cancer J Clin. 2016;66:115‐132. 26808342 10.3322/caac.21338

[4] Shaw JJ , Shah SA . Rising incidence and demographics of hepatocellular carcinoma in the USA: what does it mean? Expert Rev Gastroenterol Hepatol. 2011;5:365‐370. 21651354 10.1586/egh.11.20

[5] Inverso G , Mahal BA , Aizer AA , Donoff RB , Chau NG , Haddad RI . Marital status and head and neck cancer outcomes. Cancer. 2015;121:1273‐1278. 25524565 10.1002/cncr.29171

[6] Hinyard L , Wirth LS , Clancy JM , Schwartz T . The effect of marital status on breast cancer‐related outcomes in women under 65: a SEER database analysis. Breast. 2017;32:13‐17. 28012410 10.1016/j.breast.2016.12.008

[7] Lagergren J , Andersson G , Talbäck M , et al. Marital status, education, and income in relation to the risk of esophageal and gastric cancer by histological type and site. Cancer. 2016;122:207‐212. 26447737 10.1002/cncr.29731

[8] Baine M , Sahak F , Lin C , Chakraborty S , Lyden E , Batra SK . Marital status and survival in pancreatic cancer patients: a SEER based analysis. PLoS ONE. 2011;6:e21052. 21698253 10.1371/journal.pone.0021052 PMC3115975

[9] Jin J‐J , Wang W , Dai F‐X , et al. Marital status and survival in patients with gastric cancer. Cancer Med. 2016;5:1821‐1829. 27264020 10.1002/cam4.758 PMC4898975

[10] Chen Z , Cui J , Dai W , Yang H , He Y , Song X . Influence of marital status on small intestinal adenocarcinoma survival: an analysis of the Surveillance, Epidemiology, and End Results (SEER) database. Cancer Manag Res. 2018;10:5667‐5676. 30532589 10.2147/CMAR.S177430 PMC6241733

[11] Li Y , Zhu MX , Qi SH . Marital status and survival in patients with renal cell carcinoma. Medicine. 2018;97:16. 10.1097/MD.0000000000010385 PMC5916654 29668592

[12] Pruthi RS , Lentz AC , Sand M , Kouba E , Wallen EM . Impact of marital status in patients undergoing radical cystectomy for bladder cancer. World J Urol. 2009;27:573‐576. 19219612 10.1007/s00345-009-0380-6

[13] Abern MR , Dude AM , Coogan CL . Marital status independently predicts testis cancer survival—an analysis of the SEER database. Urol Oncol. 2012;30:487‐493. 20870430 10.1016/j.urolonc.2010.03.005

[14] El Ibrahimi S , Pinheiro PS . The effect of marriage on stage at diagnosis and survival in women with cervical cancer. Psycho‐Oncol. 2017;26:704‐710. 10.1002/pon.4070 26810012

[15] Wang FF , Xie XY , Yang XM , Jiang GQ , Gu J . The influence of marital status on the survival of patients with Hodgkin lymphoma. Oncotarget. 2017;8:51016‐51023. 28881625 10.18632/oncotarget.16879 PMC5584226

[16] Wu C , Chen P , Qian J‐J , et al. Effect of marital status on the survival of patients with hepatocellular carcinoma treated with surgical resection: an analysis of 13,408 patients in the surveillance, epidemiology, and end results (SEER) database. Oncotarget. 2016;7:79442‐79452. 27769053 10.18632/oncotarget.12722 PMC5346726

[17] Surveillance, Epidemiology, and End Results (SEER) Program . (http://www.seer.cancer.gov) SEER*Stat Database: Incidence—SEER 9 Regs Research Data, Nov 2017 Sub (1973–2015) <Katrina/Rita Population Adjustment> ‐ Linked To County Attributes ‐ Total U.S., 1969–2016 Counties, National Cancer Institute, DCCPS, Surveillance Research Program, released April 2018, based on the November 2017 submission.

[18] Jatoi A , Novotny P , Cassivi S , et al. Does marital status impact survival and quality of life in patients with non‐small cell lung cancer? Observations from the mayo clinic lung cancer cohort. Oncologist. 2007;12:1456‐1463. 18165623 10.1634/theoncologist.12-12-1456

[19] Molloy GJ , Stamatakis E , Randall G , Hamer M . Marital status, gender and cardiovascular mortality: behavioural, psychological distress and metabolic explanations. Soc Sci Med. 2009;69:223‐228. 19501442 10.1016/j.socscimed.2009.05.010 PMC2852675

[20] Goldzweig G , Andritsch E , Hubert A , et al. Psychological distress among male patients and male spouses: what do oncologists need to know? Ann Oncol. 2010;21:877‐883. 19822532 10.1093/annonc/mdp398

